# Germline deletion of Cdyl causes teratozoospermia and progressive infertility in male mice

**DOI:** 10.1038/s41419-019-1455-y

**Published:** 2019-03-08

**Authors:** Xiaoyu Xia, Xiaowei Zhou, Yanmei Quan, Yanqin Hu, Fengying Xing, Zhengzheng Li, Bufang Xu, Chen Xu, Aijun Zhang

**Affiliations:** 1 Department of Histo-Embryology, Genetics and Developmental Biology, Shanghai Jiao Tong University, School of Medicine; Shanghai Key Laboratory of Reproductive Medicine, 280 South Chongqing Road, Shanghai, 200025 China; 2 Reproductive Medical Center of Ruijin Hospital, Shanghai Jiao Tong University, School of Medicine, 197 Ruijin 2nd Road, Shanghai, 200025 China; 3 Department of Laboratory Animal Science, Shanghai Jiao Tong University, School of Medicine, 280 South Chongqing Road, Shanghai, 200025 China

## Abstract

*Chromodomain Y* (*CDY*) is one of the candidate genes for male dyszoospermia related to Y chromosome microdeletion (YCM). However, the function of CDY in regulating spermatogenesis has not been completely determined. The mouse *Cdyl (CDY-like)* gene is the homolog of human *CDY*. In the present study, we generated a germline conditional knockout (cKO) model of mouse *Cdyl*. Significantly, the *Cdyl*^*cKO*^ male mice suffered from the defects in spermatogonia maintenance and spermatozoon morphogenesis, demonstrating teratozoospermia and a progressive infertility phenotype in early adulthood. Importantly, patterns of specific histone methylation and acetylation were extensively changed, which disturbed the transcriptome in *Cdyl*^*cKO*^ testis. Our findings indicated that *Cdyl* is crucial for spermatogenesis and male fertility, which provides novel insights into the function of CDY gene, as well as the pathogenesis of YCM-related reproductive failure.

## Introduction

Since the turn of the millennium, the global incidence rate of infertility has been rising continuously. Among all infertility cases, ~50% of them can be attributed to male factors, such as idiopathic oligozoospermia or azoospermia. One of the most frequent molecular genetic causes of spermatogenic failure is deletion of the azoospermia factor region of the Y chromosome. Y chromosome microdeletion (YCM) was detected in about 2–5% of patients with severe oligozoospermia and in 5–10% of patients with azoospermia^1^. Studies on YCM are particularly important because of its potential for genetic transmission to the offspring. To date, genes including *DAZ* (deleted in azoospermia)^2^, *RBMY* (RNA binding motif protein, Y-linked)^3^, *USP9Y* (ubiquitin specific peptidase 9, Y-linked)^4^, *TSPY* (testis-specific protein, Y-linked)^5^, and *CDY* (chromodomain Y)^6^ have been identified as candidates for YCM. In contrast to the *DAZ* gene, whose critical impact upon human spermatogenesis has been fully elucidated, the functional role of the *CDY* gene cluster remains unknown. The deletion or low expression of *CDY* genes is closely correlated with male dyszoospermia^7–12^, however, the precise molecular mechanism involved remains to be investigated.

The human *CDY* gene family originated by transposition of an autosomal genomic *Cdyl* gene in primates^6,13^. In mice, there is no *CDY* gene on the Y chromosome; instead, the autosomal *Cdyl* gene is homologous to the human CDY gene family. The protein products of either human *CDY*/*CDYL* or mouse *Cdyl* genes share high similarity^14^. In the present study, we generated a *Cdyl* germline conditional knockout (*Cdyl*^*cKO*^) mouse model. The *Cdyl*^*cKO*^ male mice demonstrated a phenotype of teratozoospermia and progressive infertility. In particular, the patterns of histone methylation and acetylation were globally altered in *Cdyl*^*cKO*^ testis, which led to transcriptomic changes and various spermatogenic defects. These findings revealed an essential role of mouse Cdyl in male fertility, and provided novel insights into the mechanism of YCM-related reproductive failure.

## Materials and methods

### Animals

The mice used in this study were bred on the C57BL/6 × 129 background. All animal experiments were carried out in accordance with the guidelines for the Use of Animals in Research issued by the Shanghai Jiao Tong University, School of Medicine.

### Whole-mount immunofluorescence staining

Embryonic day 15.5 (E15.5) embryos were dissected from euthanized pregnant females, and XY embryonic gonads (EGs) were collected according a previously published method^15^. EGs were washed in phosphate-buffered saline (PBS), transferred into 4% paraformaldehyde, and fixed overnight at 4 °C on a rocker. The EGs were then subjected to three 15 min washes with PBS containing 0.1% Triton-X (PBS-T). All the antibodies were diluted in PBS-T containing 1 mg/ml bovine serum albumin (BSA). The samples were incubated with primary antibodies for 2 days at 4 °C, washed with PBS-T, and then incubated with the Alexa Fluor-conjugated secondary antibodies for 1 h at room temperature. After washing with PBS-T, the EGs were viewed and photographed under a fluorescence microscope (Leica). The antibodies used in this research are listed in Table S1.

### Generation of cKO mouse and genotyping

Conditional *Cdyl* knockout mice were generated by inserting loxP sites flanking the fifth exon of the ubiquitously expressed *Cdyl* transcript. The targeting vector was electroporated into embryonic stem cells (ESCs) to construct the heterozygous *Cdyl*^*+/flox*^ ESCs, followed by injection of the *Cdyl*^*+/flox*^ ESCs into mouse blastocysts. After the standard breeding procedures, homozygous *Cdyl*^*flox/flox*^ mice were obtained, which were phenotypically normal. The female *Cdyl*^*flox/flox*^ mice were crossed to the male *Vasa-Cre* transgenic mice to obtain the *Vasa-Cre*^+^, *Cdyl*^flox/flox^ mice (*Cdyl*^*cKO*^). Either *Cdyl*^*flox/flox*^ or *Cdyl*^*+/flox*^ male mice were used as controls (*Cdyl*^*Ctrl*^) in this study. DNA was extracted from mouse tails and subjected to PCR. The primers used to detect *Vasa-Cre*, *Cdyl*^*flox*^, *Cdyl* ^*Δ*^ (*Cdyl* deletion) in this assay are listed in Table S2.

### Mating experiment

Each *Cdyl*^*cKO*^ or control male mouse was bred with two wild-type adult females continuously from 6 weeks old. The mating experiments lasted for at least 16 weeks. The date of delivery and the numbers of litters and pups were recorded.

### RNA extraction and quantitative real-time reverse transcription PCR

Total RNA was extracted using the TRIzol reagent according to the manufacturer’s protocols (Invitrogen). Reverse transcription reactions were executed using PrimeScript RT reagent Kit (Takara). The quantitative real-time PCR reaction was prepared using the SYBR Premix Ex Taq Kit (Takara) and performed using an ABI 7500 System (Applied Biosystems). *Gapdh* was used as the internal control. The primers used in this experiment are listed in Table S2.

### Protein preparation and western blotting

To prepare the proteins from testes and mouse/human sperm, the samples were lysed in Radioimmunoprecipitation assay buffer and then homogenized on ice. The lysates were then centrifuged at 12,000 × *g* for 10 min at 4 °C to collect the supernatant. Protein concentrations were measured using a BCA Protein Assay Kit (Beyotime). All samples were processed using 10–12% sodium dodecyl sulfate polyacrylamide gel electrophoresis and electrotransferred to polyvinylidene fluoride membranes. Membranes were blocked for 1 h at room temperature with 5% BSA and then incubated with primary antibodies overnight at 4 °C. After incubation with labeled secondary antibodies, signals were obtained using a Visualizer Western Blot Detection Kit (Millipore). The primary antibodies are listed in Table S1.

### Histological and immunohistochemistry assays

Male mice were killed at 6 weeks, 14 weeks, or 5 months old. The weights of the whole body, testis, and epididymis were recorded. Tissues were fixed in Bouin’s solution overnight. Paraffin sections (5 μm) were prepared using standard procedures. Periodic acid-Schiff (PAS) staining was performed according to the manufacturer's instructions (Beyotime). Staging of mouse seminiferous tubule cross-sections was performed according to a published method^16^.

For the immunohistochemical assays, antigen retrieval was executed by incubation in buffered citrate (pH 6.0) for 15 min at 105 °C. The sections were blocked in counterpart IgG for 30 min at room temperature and then incubated with primary antibodies at 4 °C overnight. On the following day, the signals were visualized using a Histostain-Plus Kit (Life Technologies). Images were captured under a microscope BX53F (Olympus). The antibodies used in this assay are listed in Table S1.

### CASA analysis and sperm staining

The cauda epididymis was dissected from an individual mouse, and then incubated in Tyrode’s Solution (Solarbio) at 37 °C for 15 min to release the sperms. The supernatant was collected, and sperm counts and motility were evaluated using the Computer Assisted Semen Analysis (CASA) system (Hamilton). Otherwise, the supernatant was centrifuged at 1000 *g* for 15 min, the sperm pellet was resuspended in 4% paraformaldehyde, and spread on precoated slides. For morphological observation, Giemsa staining was performed according to the manufacturer's instructions (Beyotime). At least 200 sperms were recorded for each sample.

### Sex hormone measurements

The mouse sex hormone levels were measured at 5 months. The exact levels of testosterone (R&D Systems), luteinizing hormone (LH) (Shanghai Xinle Biotechnology), and follicle stimulating hormone (FSH) (Elabscience) in serum were detected using immunoassay kits according the manufacturer's protocols.

### Transmission electron microscopy

Testis tissues or sperm pellets were fixed with 2.5% glutaraldehyde in 0.2 m cacodylate buffer overnight. After washing in 0.2 m PBS, the tissues were immersed in 1% OsO_4_ in 0.2 m cacodylate buffer for 2 h at 4 °C. The samples were then dehydrated and embedded in resin. Ultrathin sections were cut and stained with uranyl acetate and lead citrate, and then observed using an H-7650 transmission electron microscope (HITACHI).

### RNA-seq library preparation and data processing

Total RNA was extracted from 6-week old testis tissues using the TRIzol reagent (Invitrogen). Complementary DNA library construction and sequencing were performed by Beijing Genomics Institute using the BGISEQ-500 platform. High-quality reads were aligned to the mouse mm10 genome using Bowtie2. The expression levels of each of gene were normalized to fragments per kilobase of exon model per million mapped reads using RNA-seq by Expectation Maximization. The interaction network of spermatogenesis-related genes was constructed by String and Cytoscape based on the published references.

### Flow cytometry

Male mice were killed at 3 weeks and their testes were harvested. A testicular cell suspension was prepared according to a previously published method^17^. After incubation with the appropriate antibodies, quantitative flow cytometry analysis was performed using bead-based 123count eBeads count (Invitrogen 01-1234-42)^18^ on CytoFlex S (Beckman). In brief, the testicular cells harvested from 3-week-old mouse were resuspended in 100 μL of PBS. Then 100 μL of beads, ~1010 beads/μL, was added to each sample. The final volume in the tube was 200 μL. Each tube was fully vortexed before collection to ensure equal dispersion of cells and beads. In each analysis, ~10,000 beads events were collected and the results were analyzed using CytExpert2.0. The absolute enumeration of Thy1 or c-Kit positive cells per mouse was calculated according to the relative ratio to beads.

### Statistical analysis

Statistical analysis was performed using one-way analysis of variance, followed by Student’s *t* test. Values of *p* *<* 0.05 were considered statistically significant. The statistical graphs were generated using GraphPad Prism 6.

## Results

### Generation of *Cdyl* germline cKO mice

**Fig. 1:**
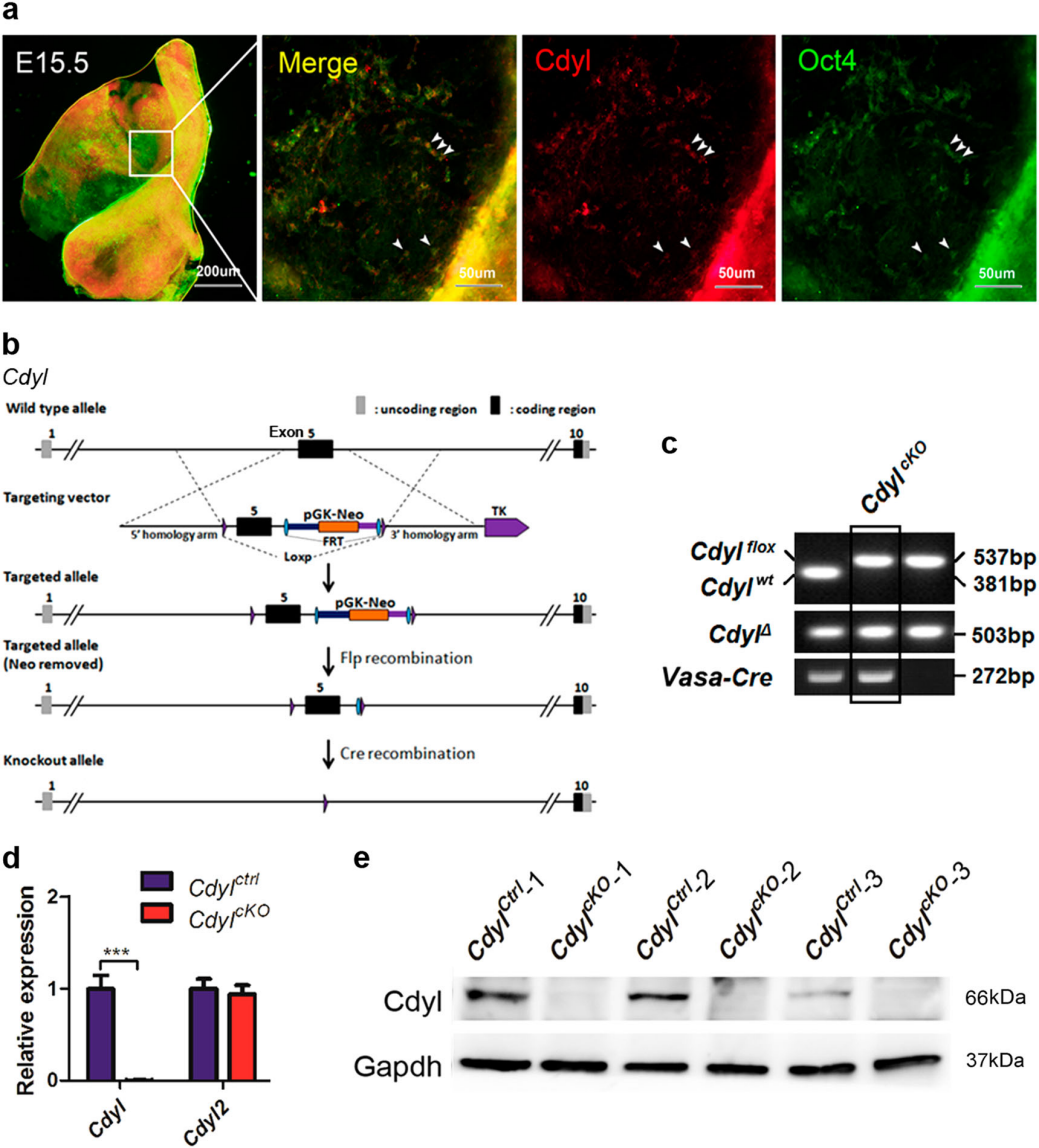
Generation of germline *Cdyl*^*cKO*^ mice **a** Whole-mount staining of Cdyl and Oct4 in mouse E15.5 male embryonic gonads. **b** Schematic overview of the gene targeting strategy to generate *Cdyl*^*cKO*^ mice. **c** Representative result of genotyping to identify the *Cdyl*^*cKO*^ mice. *Cdyl*^*flox*^, loxP flanked allele; *Cdyl*^*wt*^, wild-type allele; *Cdyl*^*Δ*^, *Cdyl* conditional deletion. **d** Verification of the conditional knockout efficiency by detection of the relative mRNA expression of *Cdyl* in *Cdyl*^*cKO*^ testis (*n* = 4). ***p *<* 0.001. **e** Verification of the conditional knockout efficiency by detecting the protein expression of Cdyl in *Cdyl*^*cKO*^ testis (*n* = 3)

The mouse *Cdyl* gene produces a longer ubiquitously expressed transcript and a shorter testis-specific transcript after birth^19^. However, it is not clear whether *Cdyl* is expressed during embryonic gonadal development. By whole-mount immunofluorescence staining, we detected the co-localization of Cdyl and octamer-binding protein 4 (Oct4) in E15.5 male mouse embryonic gonads (Fig. 1a, arrowhead). As Oct4 is the acknowledged marker of primordial germ cells (PGCs)^20^, it was suggested that Cdyl also functions in mouse male PGCs.

In the present study, male *Vasa-Cre* transgenic mice were used to generate *Cdyl* germline cKO mice, considering that the recombinase would be active in *Vasa-Cre*^*+*^ germ cells as early as E15^21^. In detail, the fifth exon of the ubiquitously expressed *Cdyl* transcript, which was identical to exon 2 in the testis-specific transcript, was flanked by inserted loxP sites. Homozygous *Cdyl*^*flox/flox*^ mice were obtained and were physiologically normal, then the female *Cdyl*^*flox*/*flox*^ mice were crossed to the male *Vasa-Cre* transgenic mice. Eventually, we successfully established the male germline cKO *Vasa-Cre*^+^,*Cdyl*^*flox/flox*^ (*Cdyl*^*cKO*^) mice (Fig. 1b, c). The results of quantitative real-time reverse transcription polymerase chain reaction (qRT-PCR) and western blotting (Fig. 1d, e) showed that the expression level of *Cdyl* was dramatically decreased in the testis of 6-week-old *Cdyl*^*cKO *^mice.

### Germline knockout of *Cdyl* caused a severe progressive infertility in male mice

We then assessed the reproductive performance of male *Cdyl*^*cKO*^ mice. Six-week-old *Cdyl*^*cKO*^ mice were bred with adult wild-type females for continuous 16 weeks (*n* = 5). The numbers of litters and pups from each tested male were recorded, as well as the exact age by which they gave the first and the last litters during the experiment (Table 1). The average litter size from the *Cdyl*^*cKO*^ males was not changed (*P* > 0.05); however, the total number of litters produced by the *Cdyl*^*cKO*^ group was only 42.86% of that from the control group (*P* *<* 0.001). The average age of *Cdyl*^*cKO*^ males that delivered their first litter was apparently older than that of the control males (*P* *<* 0.05). In addition, their average age of delivery of the last litter was around 15 weeks old, whereas the control males remained fertile for at least 10 months (*P* *<* 0.01).

**Table 1:** The fertility of *Cdyl*^*cKO*^ male mice

	*Cdyl*^*Ctrl*^ (*n* = 5)	*Cdyl*^*cKO*^ (*n* = 5)	*P*
Age of first litter (PND)	64.2 ± 8.70	76.2 ± 8.07	<0.05
Age of last litter (PND)	142.4 ± 12.29	96 ± 15.95	<0.01
Total litters	21	9	
Average litters	4.2 ± 0.45	1.8 ± 0.45	<0.001
Total pups	150	58	
Average litter size	7.14 ± 2.52	6.44 ± 2.69	>0.05

*Cdyl*^*cKO*^ mice were bred to adult wild-type females for continuous 16 weeks, the numbers of litters and pups from each tested male were recorded (*n* = 5). Values represent the mean ± SEM; statistical analyses were conducted using Student’s *t* test. PND, post-natal day

**Fig. 2:**
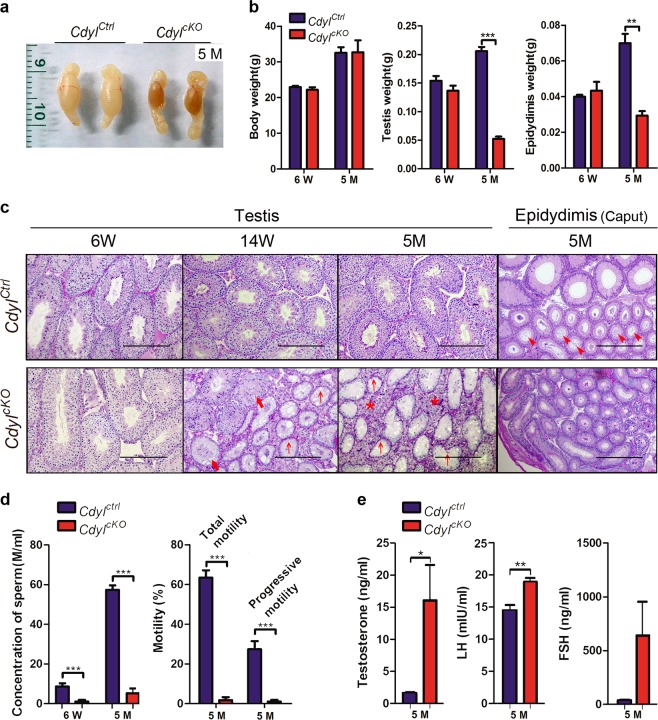
*Cdyl*^*cKO*^ male mice exhibited the progressive infertility **a** Testes harvested from *Cdyl*^*Ctrl*^ and *Cdyl*^*cKO*^ mice at 5 months. **b** Weight of body, testis, and epidydimis in *Cdyl*^*Ctrl*^ and *Cdyl*^*cKO*^ group (*n* = 6). **c** Pathology of *Cdyl*^*cKO*^ testis at 6 weeks, 14 weeks, and 5 months; Pathology of *Cdyl*^*cKO*^ epidydimis at 5 months. Bar = 200 μm. Bold arrow, loss of spermatogenic cells; arrow, Sertoli-cell-only syndrome phenotype; asterisk, hyperplasia of the interstitial tissues; arrowhead, seminal fluid in the lumen of epididymis. **d** Computer Assisted Semen Analysis of *Cdyl*^*cKO*^ males at 6 weeks and 5 months (*n* = 6). **e** Serum sex hormone levels of *Cdyl*^*cKO*^ males at 5 months (*n* = 5). *p *<* 0.05; **p *<* 0.01; ***p *<* 0.001

By 5 months after birth, the testis from *Cdyl*^*cKO*^ mice had a shrunken and sclerotic appearance (Fig. 2a). Although there was no difference in body weight, the weight of the testis or epididymis was significantly reduced compared with those of the wild-type (*n* = 6) (Fig. 2b). Histopathological examination revealed that a progressive degeneration of spermatogenesis happened in *Cdyl*^*cKO*^ testis from 6 weeks until 5 months (Fig. 2c); the germ cells were gradually lost in most of the seminiferous tubules (bold arrow). We observed a typical phenotype of Sertoli-cell-only syndrome (SCOS)^22^ in 5-month-old *Cdyl*^*cKO*^ testis (arrow), that only Sertoli cells were lining the seminiferous tubules. Meanwhile, hyperplasia of the interstitial tissues was noticed (asterisk). Usually, the lumen of epididymis is full of the seminal fluid in adult mice (arrowhead), however, only a few spermatozoa were found in epididymal sections of 5-month-old *Cdyl*^*cKO*^ males.

Along with these findings, the CASA analysis disclosed a decrease in the sperm concentration in *Cdyl*^*cKO*^ male mice. Compared with that in the control group, it was only about 18.88% (1.62 ± 0.50 m/ml vs. 8.58 ± 3.18 m/ml) in 6-week-old *Cdyl*^*cKO*^ males, and then declined to 8.99% (5.16 ± 6.15 m/ml vs. 57.34 ± 5.19 m/ml) by 5 months (*n* = 6) (Fig. 2d). We next measured the serum sex hormone levels in 5-month-old *Cdyl*^*cKO*^ male mice (*n* = 5). We observed upregulation of testosterone (*P* *<* 0.05) and LH (*P* *<* 0.01), and a non-significant increase in FSH (*n* = 5) (Fig. 2e). In conclusion, the *Cdyl*^*cKO*^ male mice suffered from the progressive infertility and displayed a severe oligozoospermia phenotype by 5 months.

### Abnormal spermiogenesis in *Cdyl*^*cKO*^ male mice

**Fig. 3:**
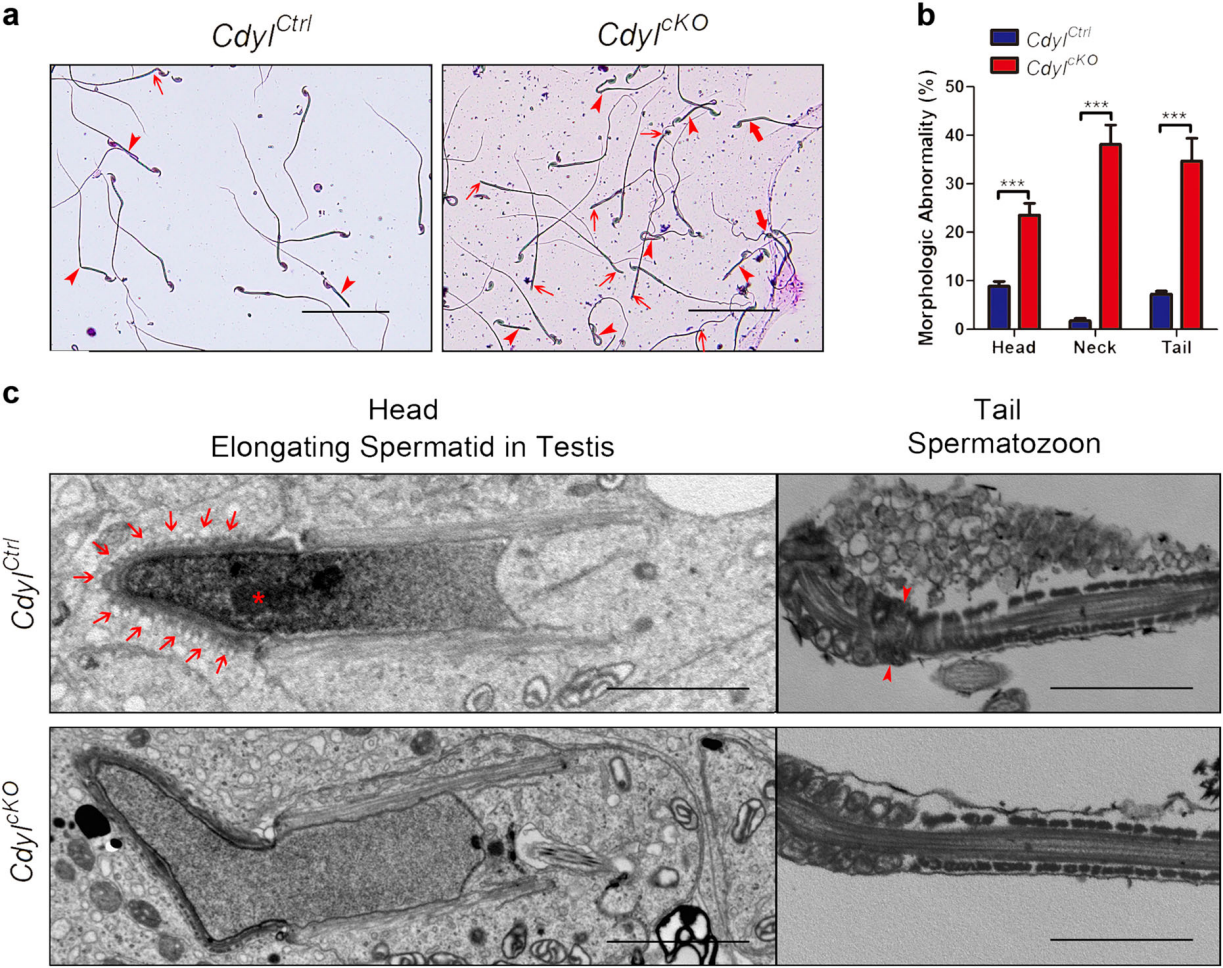
Abnormal morphogenesis of spermatozoon in *Cdyl*^*cKO*^ mice **a** Morphology of spermatozoa from 14-week-old *Cdyl*^*cKO*^ male mice under light microscopy. Bar = 200 μm. Bold arrow, abnormal shape of head; arrow, detached head due to the faulted neck structure; arrowhead, bending or looping of flagellum. **b** Percentage of sperm malformation in 14-week-old *Cdyl*^*cKO*^ male mice (*n* = 5). ***p < 0.001. **c** Morphology of spermatid and spermatozoa from 14-week-old *Cdyl*^*cKO*^ male mice under transmission electron microscopy. Bar = 2 μm. Asterisk, organization center of nuclear concentration; arrow, ectoplasmic specialization; arrowhead, annulus structure

In addition to the quantitative loss of sperm, the total and progressive sperm motilities were also badly damaged in 5-month-old *Cdyl*^*cKO*^ males (*P* *<* 0.001) (Fig. 2d). We also observed malformation of spermatozoon in *Cdyl*^*cKO*^ mice (Fig. 3a, b), for example, the abnormal shape of head (bold arrow), detached head caused by a faulty neck structure (arrow), and bending or looping of flagellum (arrowhead) (*n* = 5, *P* *<* 0.001). Many sperms carried head and tail defects at the same time.

Using transmission electron microscopy, we inspected the ultrastructure of the spermatids in testis sections or the spermatozoon extracted from the cauda epididymis (Fig. 3c). Compared with the control samples, many of the elongating spermatids from *Cdyl*^*cKO*^ male lacked the organization center of nuclear concentration (asterisk), which was replaced by homogenous diffused nucleoplasm. Ectoplasmic specialization (arrows), the particular cell junctions that connect the spermatids and Sertoli cells^23^, were also disrupted in *Cdyl*^*cKO*^ testis. In another aspect, the annulus structure (arrowhead) partitions the middle piece and the principle piece along the sperm tail^24^. However, this structure was frequently missing in the *Cdyl*^*cKO*^ spermatozoa, contributing to the bending of the flagellum at that exact spot. In summary, the conditional deletion of *Cdyl* might lead to the incorrect morphogenesis of spermatozoon and a teratozoospermia phenotype.

### *Cdyl* cKO influenced the patterns of histone modifications in testis

**Fig. 4:**
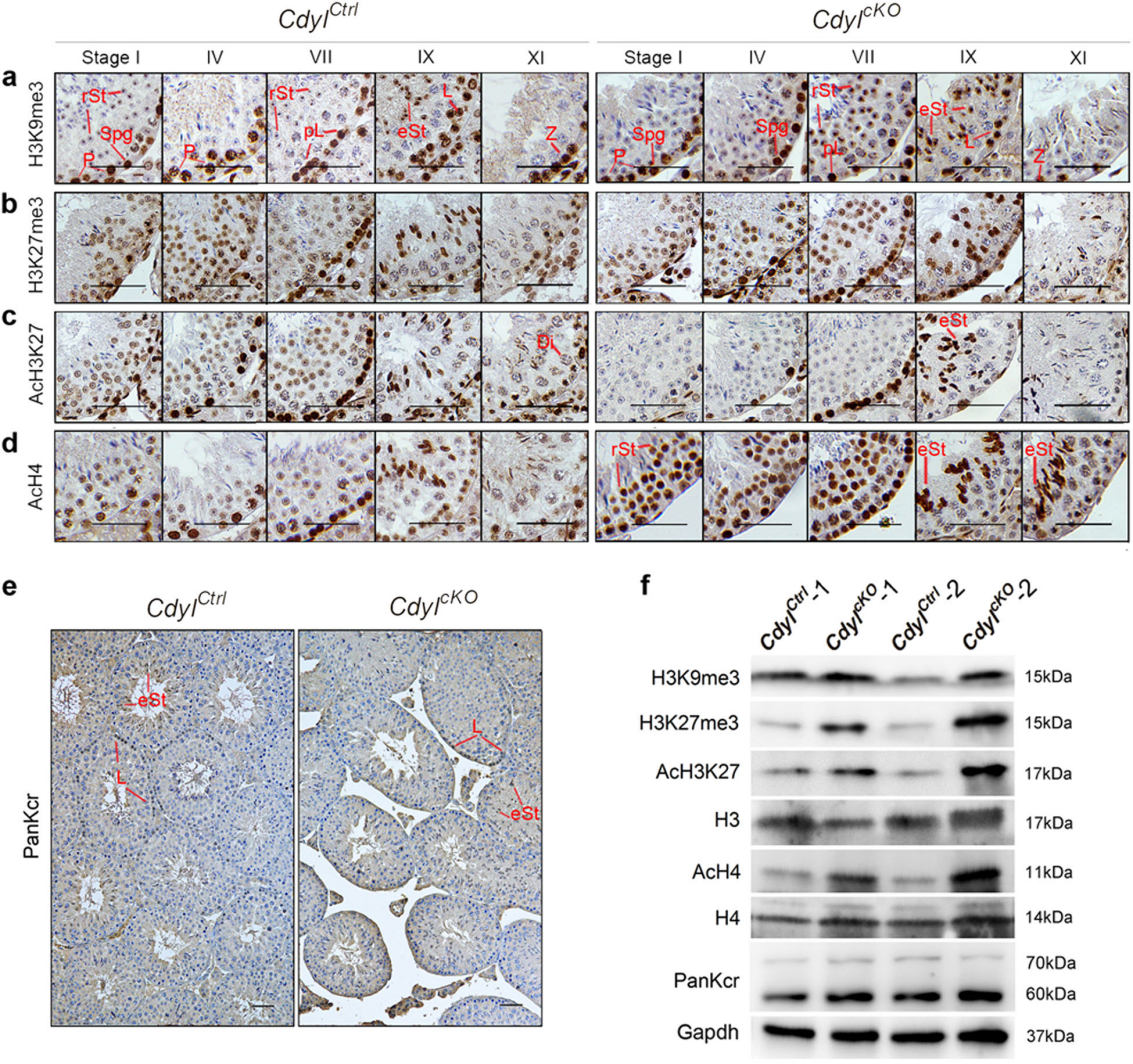
Histone modification patterns were disturbed in *Cdyl*^*cKO*^ testis by 6 weeks Immunostaining of **a** H3K9me3; **b** H3K27me3; **c** AcH3K27; **d** AcH4; **e** PanKcr in *Cdyl*^*cKO*^ testis. Bar = 50 μm. Classification of stage I, IV, VII, IX, XI seminiferous tubules was performed according to a published method^16^. Spg, spermatogonia; pL, preleptotene spermatocytes; L, leptotene spermatocytes; Z, zygotene spermatocytes; P, pachytene spermatocytes; Di, diplotene spermatocytes; rSt, round spermatids; eSt, elongating spermatids. **f** Expression level of given histone modifications in *Cdyl*^*cKO*^ testis by western blotting detection

As shown above, the *Cdyl* cKO had complicated consequences for mouse spermatogenesis. The results implied that Cdyl has important roles in multiple ways and stages during this process. To date, our knowledge of CDYL/Cdyl proteins has focused on their functions in epigenetic regulation, especially histone methylation^25–27^ and crotonylation^28^. According to these reports, we evaluated the histone H3 lysine 9 trimethylation (H3K9me3) expression in 6-week-old testicular sections (Fig. 4a). In the normal control group, a strong nuclear signal of H3K9me3 was present in spermatogonia and middle-pachytene spermatocytes, and then disappeared in the late-pachytene phase. After meiosis, H3K9me3 formed a condensed spot in round spermatid nuclei, persisting until step 9–10 elongating spermatids. In comparison, the H3K9me3 signal almost vanished in the pachytene spermatocytes of the *Cdyl*^*cKO*^ testis; however, it became much stronger than that in the control group through step 6–11 of spermiogenesis.

We then examined the expression of histone H3 lysine 27 trimethylation (H3K27me3). In control tissues, a modest H3K27me3 signal was detected in all stages of spermatogenic cells, reaching a peak in leptotene spermatocytes and elongating spermatids (Fig. 4b). However, the H3K27me3 signal in the *Cdyl*^*cKO*^ testis specifically increased in intermediate/type B spermatogonia, as well as in step 7–11 spermatids undergoing transformation. In contrast, we detected a fluctuating pattern of the acetylated histone H3 lysine 27 (AcH3K27) signal in the *Cdyl*^*cKO*^ testis. Compared with that in the control group, the AcH3K27 signal accumulated in leptotene spermatocytes, but gradually weakened in later spermatocytes and round spermatids, and increased again in elongating spermatids (Fig. 4c). We next studied the expression of acetylated histone H4 (AcH4). In spermatogonia and spermatocytes, the AcH4 signal was indistinctive to that in control samples. Surprisingly, in the majority of the spermatids, including the early round and later elongating ones, the AcH4 signal was markedly elevated (Fig. 4d). Recently, human CDYL was reported to be a negative regulator of histone lysine crotonylation, and overexpressing *Cdyl* caused male subfertility in mice^28^. Therefore, we tested the global histone crotonylation (PanKcr) level in 6-week-old *Cdyl*^*cKO*^ testis. Unexpectedly, the signal of PanKcr was not affected (Fig. 4e).

Finally, we checked the overall protein levels of the above histone modifications using western blotting (Fig. 4f). Except for PanKcr, all the other histone modifications detected were upregulated in 6-week-old *Cdyl*^*cKO*^ testis. Taken together, we revealed that *Cdyl* conditional deletion resulted in disrupted patterns of histone modification.

### *Cdyl* conditional deletion disturbed the transcriptome in the testis

**Fig. 5:**
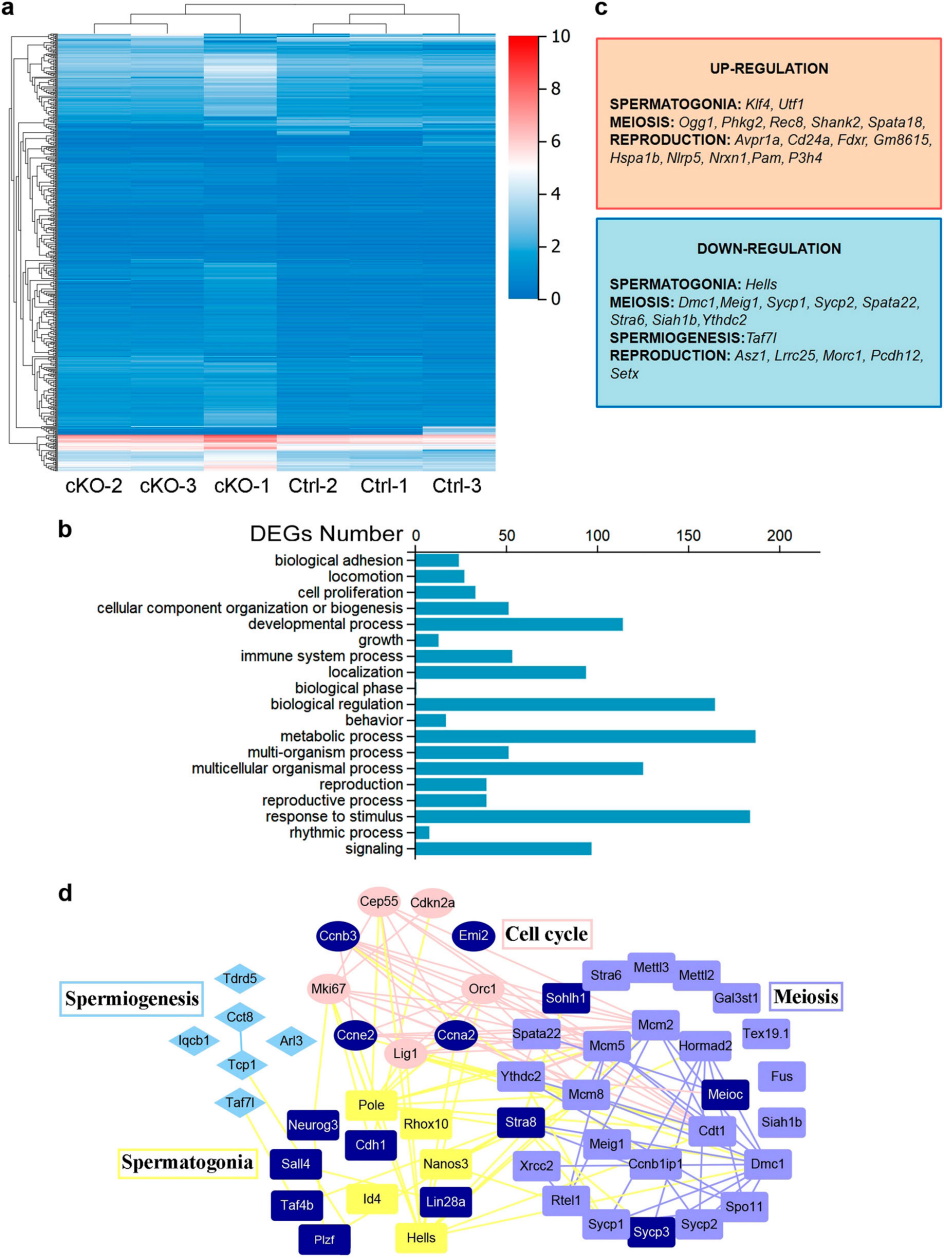
*Cdyl* conditional deletion altered the transcriptome in *Cdyl*^*cKO*^ testis by 6 weeks **a** Heatmap showing the differentially expressed genes between *Cdyl*^*Ctrl*^ and *Cdyl*^*cKO*^ testes (*n* = 3). Twofold expression difference and adjust *p* ≤   0.01 were used as the cutoff. **b** The most affected gene ontology (GO) Terms in *Cdyl*^*cKO*^ testis. **c** Representatives of the dysregulated spermatogenesis-related genes in *Cdyl*^*cKO*^ testis. **d** Interaction network showing the dysregulated genes in *Cdyl*^*cKO*^ testis that involved in spermatogenesis regulation. The genes validated by quantitative real-time reverse transcription polymerase chain reaction (qRT-PCR) are marked in dark blue

Transcriptional activity is dynamically regulated by epigenetic modifications, including histone methylation and acetylation. Therefore, we compared the transcriptome between the 6-week-old *Cdyl*^*cKO*^ testis and their control counterparts using high-throughput RNA sequencing (RNA-seq) (*n* = 3 each). We identified 675 differentially expressed genes (DEGs) in the *Cdyl*^*cKO*^ group (Fig. 5a). These DEGs were enriched in gene clusters of the cell proliferation, developmental process, and reproduction (Fig. 5b, c). Genes with functions in spermatogenesis^29,30^ were mapped into an interaction network using String and Cytoscape (Fig. 5d). In particular, we checked the sex chromosome-linked genes that reported highly expressed in round spermatids and are regulated by histone crotonylation modification^28^. However, no significant change was found in the expression of these genes from the RNA-seq analysis (Supplementary Table S3).

**Fig. 6:**
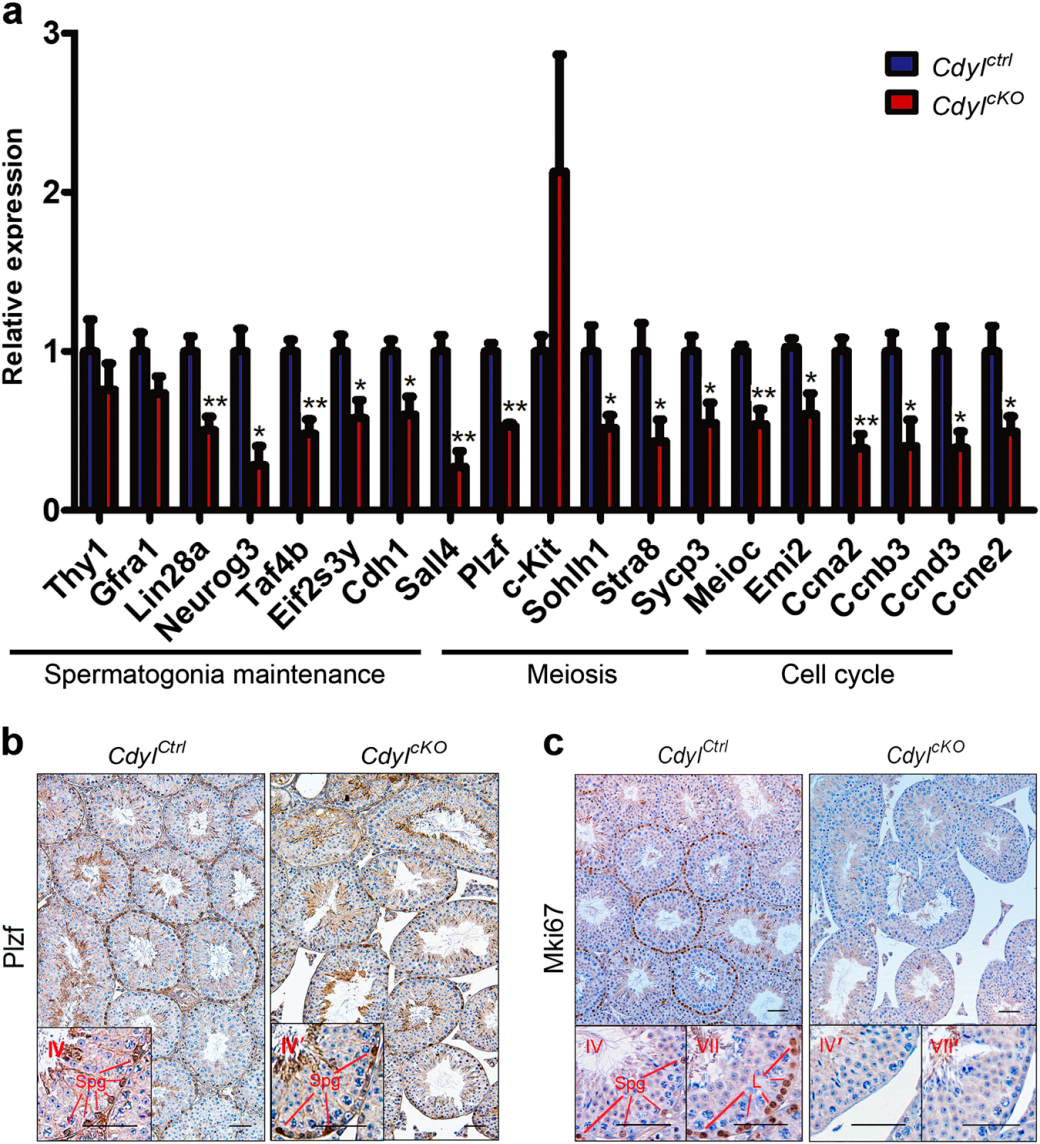
Expression of spermatogenesis-related genes was affected by *Cdyl* conditional deletion **a** Quantitative real-time reverse transcription polymerase chain reaction (qRT-PCR) validation of dysregulated genes involved in spermatogenesis in *Cdyl*^*cKO*^ testis (*n* ≥ 3). *p *<* 0.05; **p *<* 0.01. **b** Staining of spermatogonial marker Plzf in *Cdyl*^*cKO*^ testis at 6 weeks. Bar = 50 μm. Spg, spermatogonia; L, leptotene spermatocytes; **c** Staining of proliferative marker Mki67 in *Cdyl*^*cKO*^ testis at 6 weeks. Bar = 50 μm

We then validated the RNA-seq results using qRT-PCR (*n* ≥ 3) (Fig. 6a). Among the target genes, *Thy1* (Thy1 cell surface antigen), *Gfra1* (GDNF family receptor alpha 1), *Lin28a* (Lin-28 homolog A), *Neurog3* (neurogenin 3), *Taf4b* (TATA-box binding protein associated factor 4b), *Eif2s3y* (Eukaryotic translation initiation factor 2 subunit 3, Y-linked), *Cdh1* (cadherin 1), *Sall4* (spalt like transcription factor 4), and *Plzf* (promyelocytic leukemia zinc finger protein) have been reported to be expressed sequentially in testis, playing crucial roles for spermatogonial stem cell (SSCs) and spermatogonia maintenance^29,31–34^. Our results revealed that most of the tested genes were downregulated in *Cdyl*^*cKO*^ testis. In another aspect, c-*Kit* (Kit proto-oncogene receptor tyrosine kinase), *Sohlh1* (spermatogenesis and oogenesis specific basic helix-loop-helix 1), *Stra8* (stimulated by retinoic acid 8), *Sycp3* (synaptonemal complex protein 3), and *Meioc* (meiosis specific with coiled-coil domain) are involved in spermatogonial differentiation or meiosis progression^32,35–37^. Generally, these genes were downregulated in *Cdyl*^*cKO*^ testis, except for c-*Kit*, which presented dramatic individual differences. For cell cycle regulator genes, *Emi2* (Endogenous meiotic inhibitor 2), *Ccna2* (cyclin A2), *Ccnb3* (cyclin B3), *Ccnd3* (cyclin D3), and *Ccne2* (cyclin E2)^38,39^ were all obviously downregulated in *Cdyl*^*cKO*^ testis. Thus, *Cdyl* conditional deletion significantly affected the testicular transcriptome, especially the expression of spermatogenesis-related genes.

This alteration of the transcriptome would profoundly interrupt the normal progress of spermatogenesis. Using immunohistochemistry, we investigated the protein levels of spermatogonial marker Plzf^40^ and proliferative marker Mki67 (marker of proliferation Ki-67)^41^. In detail, there were far fewer Plzf positive spermatogonia in 6-week-old *Cdyl*^*cKO*^ testis compared with that in the control (Fig. 6b), implying deficiency of spermatogonia maintenance. In the 6-week-old control samples, a strong Mki67 signal was detected in all stages of spermatogonia, as well as in the daughter leptotene spermatocytes. Surprisingly, Mki67 staining completely disappeared in the *Cdyl*^*cKO*^ testis, indicating the impediments of spermatogonial mitosis (Fig. 6c).

### Progressive infertility of *Cdyl*^*cKO*^ males owing to the deficiency in spermatogonia maintenance

**Fig. 7:**
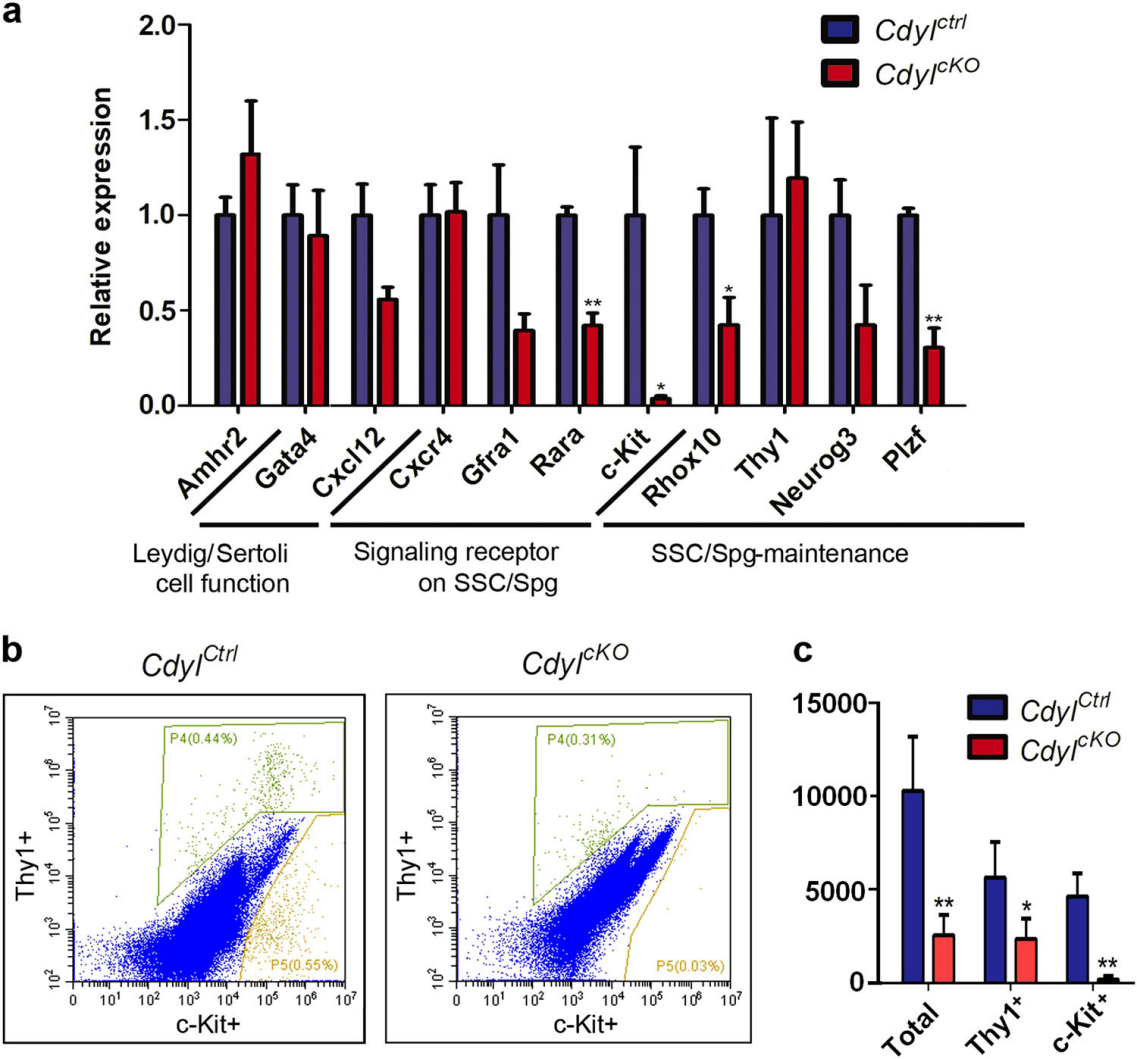
Spermatogonia maintenance was disrupted in *Cdyl*^*cKO*^ male mice **a** Relative expression of important marker genes in 1-week-old *Cdyl*^*cKO*^ testis (*n* = 3). SSC, spermatogonial stem cell; Spg, spermatogonia. **b** Representative histogram of quantitative flow cytometry for SSCs/ spermatogonia identification. The Thy1^+^ events were gated as P4, while the c-Kit^+^ events were gated as P5. **c** Changes in the absolute number of SSCs/ spermatogonia in *Cdyl*^*cKO*^ males by 3 weeks (*n* = 4). *p *<* 0.05; **p *<* 0.01

As shown in Fig. 6b, c, spermatogonial maintenance was already disrupted in *Cdyl*^*cKO*^ testis by 6 weeks old. Therefore, we collected the testis from 1 week after birth, when meiosis has not started, and only SSCs/spermatogonia and Sertoli cells were present in the seminiferous tubules^42^. *Amhr2* (anti-mullerian hormone receptor type 2) is the functional marker of Leydig cells^43^, whereas *Gata4* (GATA-binding protein 4) and *Cxcl12* (C-X-C motif chemokine ligand 12) are markers of Sertoli cells^29,44,45^. Using qRT-PCR (Fig. 7a), we detected no differences in the expression levels of *Amhr2*, *Gata4*, and *Cxcl12* in *Cdyl*^*cKO*^ samples. We then evaluated the critical signaling receptors expressed by SSCs/spermatogonia. Significantly, the RA receptor *Rara*^46^ and the SCF receptor *c-Kit* were downregulated, and the same inclination was observed for the GDNF family receptor *Gfra1*. Meanwhile, we observed decreased expression levels of *Rhox10* (reproductive homeobox 10)^47^ and *Plzf*, which are essential for mouse SSC establishment.

Furthermore, we analyzed the absolute numbers of SSCs/spermatogonia in 3-week-old testis (*n* = 4) using quantitative flow cytometry (Fig. 7b, c, Figure S1). Thy1 has been recognized as a marker of undifferentiated spermatogonial stem cells^48^, whereas c-Kit is a hallmark for differentiating spermatogonia^49^. Strikingly, the absolute number of Thy1^+^ cells or c-Kit^+^ cells was much lower in the *Cdyl*^*cKO*^ group (*P* *<* 0.05, *P* *<* 0.01, individually). Thus, the total amount of SSCs/spermatogonia was remarkably reduced in *Cdyl*^*cKO*^ testis by 3 weeks old (*P* *<* 0.01).

## Discussion

Although the human *CDY* gene has been identified as a candidate of YCM for decades, the biological functions of CDY or its autosomal homologs have not been thoroughly resolved^6^. Both human CDY/CDYL and mouse Cdyl proteins comprise an N-terminal chomodomain and a C-terminal crotonase-like fold^14^. Based on the properties of chromodomains, CDYL/Cdyl has been proven as an H3K9me3 and H3K27me3 reader, functioning as the mediator in various transcriptional repressive complexes^26,27,50^. This co-repressor effect of CDYL/Cdyl is important in neural development^51–54^, X chromosome inactivation^55^, and even the transformation of tumor cells^26^. Furthermore, human CDYL plays a vital role in the transmission/restoration of repressive histone markers and the maintenance of cell identity^56^.

However, the molecular functions of CDY/CDYL/Cdyl in spermatogenesis are not fully understood. Lahn et al.^19^ reported that, mouse Cdyl was co-localized with the acetyl-H4 in developing spermatids, possibly functioning as a histone acetyltransferase in spermatogenesis. This assumption was disproved by Franz et al.^57^, who failed to reproduce the histone acetyltransferase activity of CDY family proteins. Recently, Liu et al.^28^ demonstrated that human CDYL negatively regulates histone lysine crotonylation (Kcr) by acting as a crotonyl-CoA hydratase. In addition, using the overexpression technique, they verified the male subfertility in mice caused by the global upregulation of Cdyl, showing the importance of Cdyl in spermatogenesis. However, there had been no ideal animal model for *CDY* deletion in some of the human YCM cases.

To clarify the authentic function of CDY/CDYL/Cdyl in spermatogenesis and male reproduction, we established the *Cdyl* germline cKO mouse model. Compared with control littermates, the time span of fertility was significantly shortened in *Cdyl*^*cKO*^ males: All the *Cdyl*^*cKO*^ males we examined developed severe oligozoospermia by 5 months (*n* ≥ 15). This peculiar phenotype of progressive infertility was also observed in *Map7* (microtubule associated protein 7)^58^, *ERM* (ermin)^59^, and *Taf4b*^60^-null mouse models, as well as in *Rhox10*^47^ cKO mice. In addition, the abnormal morphogenesis of spermatozoon was observed in *Cdyl*^*cKO*^ males (Fig. 3), resembling human teratozoospermia and asthenozoospermia.

Importantly, we detected disordered patterns of given histone methylation and acetylation in *Cdyl*^*cKO*^ testis (Fig. 4a–d), especially in haploid spermatids, where epigenetic reprogramming is active^61^. This suggested that mouse Cdyl is indispensable for the control of spermiogenesis, even the setups of paternal epigenetic imprinting. Among these histone modifications, H3K9me3 and H3K27me3 are usually recognized as markers of heterochromatin, whereas AcH3K27 and AcH4 indicate the open configuration of chromatin. The broad alteration of histone modifications would have an extensive impact on histone dynamics and chromatin architecture, and ultimately, transcriptional activity. As a result, the transcriptome was disrupted in *Cdyl*^*cKO*^ testis, and the affected genes included those involved in SSC maintenance, spermatogonial differentiation, meiosis progression, spermiogenesis, and cell cycle control (Figs. 5, 6), which in turn led to the more complex spermatogenic defects. It put forward the question that, whether the inhibitors of histone methyltransferase and/or acetylase, such as G9a inhibitor UNC0642 or EZH2 inhibitor UNC1999 would rescue the phenotype of dyszoospermia in *Cdyl*^*cKO*^ testis. In our study, global histone crotonylation seemed not affected by *Cdyl* germline cKO (Fig. 4e, f); therefore, phase and site-specific histone crotonylation should be analyzed.

In rodent testis, there are two distinct subsets of spermatogonia: one remains undifferentiated and establishes the SSC pool throughout adulthood; and the other differentiates directly to initiate the first wave of spermatogenesis^62,63^. By quantitative flow cytometry, we proved the decline in undifferentiated spermatogonia caused by *Cdyl* conditional deletion (Fig. 7c). It reflected the defects in the establishment and maintenance of the proliferative spermatogonia, which would lead to the exhaustion of spermatogonia and eventual infertility in *Cdyl*^*cKO*^ mice. It could be the consequence of the intrinsic failure of SSC self-renewal, as well as the damaged crosstalk between the spermatogenic cells and their niche. To testify these hypotheses, spermatogonia could be enriched from *Cdyl*^*cKO*^ testis and transplanted into the recipient seminiferous tubules, by which the role of Cdyl in spermatogenesis would be utterly evaluated. Since our data indicated the mouse Cdyl was expressed in E15.5 PGCs (Fig. 1a), and the disrupted transcription happened as early as 1-week old (Fig. 7a), the defects occurring in *Cdyl*^*cKO*^ testis probably originated during the phase of embryonic gonads. Ongoing work in our laboratory is to trace the developmental trajectory of PGCs in *Cdyl*^*cKO*^ mice.

In humans, there are Y chromosome-specific *CDY* genes as well as autosomal *CDYL* genes. Current evidences showed that the deletion or low expression of *CDY* genes correlate with the human spermatogenic defects. In some cases, spermatogenesis was blocked at the stage of spermatid maturation, in spite of the existence of spermatocytes^8,10,12^. We supposed that the CDYL and CDY proteins could compensate mutually in human testis. Only if both of them were underexpressed would the SCOS phenotype develop, as observed in the *Cdyl*^*cKO*^ mouse model. In summary, we revealed the comprehensive effect of mouse Cdyl on spermatogenesis in vivo. Our findings will aid the mechanism research and potential therapy for the human CDY-associated dyszoospermia.

## Supplementary information

Supplementary Table S1

Supplementary Table S2

Supplementary Table S3

Supplementary Figure S1

Supplementary Figure S2

supplemental figure legends
