# Supplementary material for: Germline deletion of Cdyl causes teratozoospermia and progressive infertility in male mice
Source: Cell Death Dis. 2019 Mar 8;10(3):229. doi: 10.1038/s41419-019-1455-y (PMC6408431; doi:10.1038/s41419-019-1455-y)
Supplement: Supplementary file 1 — Supplementary Table S1 [file 41419_2019_1455_MOESM1_ESM.docx]

**Supplementary Table 1** Antibodies used in this study.

| **Antibodies** | **Catalogue number** | **Company** |
| --- | --- | --- |
|  |  |  |
| **Rabbit anti-Cdyl/ CDYL** | NBP2-15851 | Novusbio |
|  |  |  |
|  |  |  |
| **Goat anti-Oct4** | ab27985 | Abcam |
| **Rabbit anti-Gapdh** | CST5174 | CST |
| **Rabbit anti-H3K9me3** | A2360 | abclonal |
| **Rabbit anti-H3K27me3** | ab192985 | Abcam |
| **Rabbit anti-AcH3K27** | ab4729 | Abcam |
| **Rabbit anti-AcH4** | ab177790 | Abcam |
|  |  |  |
| **Rabbit anti-PanKcr** | PTM-501 | PTM BIO |
| **Rabbit anti-H3** | ab1791 | Abcam |
| **Rabbit anti-H4** | ab10158 | Abcam |
| **Rabbit anti-Plzf** | sc-22839 | Santa Cruz |
| **Rabbit anti-Mki67** | sc-15402 | Santa Cruz |
| **Rat anti-Thy1-PE/CY5.5** | ab25272 | Abcam |
| **Rat anti-c-Kit-FITC** | ab24870 | Abcam |
|  |  |  |
