# Supplementary material for: Germline deletion of Cdyl causes teratozoospermia and progressive infertility in male mice
Source: Cell Death Dis. 2019 Mar 8;10(3):229. doi: 10.1038/s41419-019-1455-y (PMC6408431; doi:10.1038/s41419-019-1455-y)
Supplement: Supplementary file 2 — Supplementary Table S2 [file 41419_2019_1455_MOESM2_ESM.docx]

**Supplementary Table 2** Primers used in this study.

| **Gene** | **Forward Primer 5’-3’** | **Reverse Primer 3’-5’** |
| --- | --- | --- |
|  |  |  |
| **Genotyping** |  |  |
| ***Vasa-Cre*** | CACGTGCAGCCGTTTAAGCCGCGT | TTCCCATTCTAAACAACACCCTGAA |
| ***Cdyl^flox^*** | CTCCCTAAACAGTCTGGTGGTATT | CAGGCAAGCATTAAAGTGTGAT |
| ***Cdyl-conditional deletion*** | GGCCACAGGCTTAGCTGTTA | CTCACACTGAAACGCAACCG |
|  |  |  |
| **qRT-PCR** |  |  |
| ***Cdyl*** | TGTGATGTGGTTTGGGCTAA | AGCAGCCATCTGGACTCTGT |
| ***Thy1*** | TGCTCTCAGTCTTGCAGGTG | TGGATGGAGTTATCCTTGGTGTT |
| ***Gfra1*** | CACTCCTGGATTTGCTGATGT | AGTGTGCGGTACTTGGTGC |
| ***Lin28a*** | TGGTGTGTTCTGTATTGGGAGT | AGTTGTAGCACCTGTCTCCTTT |
| ***Neurog3*** | CCAAGAGCGAGTTGGCACT | CGGGCCATAGAAGCTGTGG |
| ***Taf4b*** | TTGCAGCTATTGGACCAAGGA | GTGGCTGTTAGGCTGGAAGT |
| ***Eif2s3y*** | ATCTTGTCCTCAACCTCAGACT | TTCTTTAGCCTGGCTTTCTTTCA |
| ***Cdh1*** | CAGGTCTCCTCATGGCTTTGC | CTTCCGAAAAGAAGGCTGTCC |
| ***Sall4*** | CCCTGGGAACTGCGATGAAG | TCAGAGAGACTAAAGAACTCGGC |
| ***Plzf*** | CTGCGGAAAACGGTTCCTG | GTGCCAGTATGGGTCTGTCT |
| ***c-Kit*** | CTCCCCCAACAGTGTATTCAC | TAGCCCGAAATCGCAAATCTT |
| ***Sohlh1*** | AGAGCGCGTTGTCATTCAGT | CATGCCGGAAGATTCAGGG |
| ***Stra8*** | TTTGACGTGGCAAGTTTCCTG | TAACACAGCCAAGGCTTTTGA |
| ***Sycp3*** | GCAGCAAGAGATGGCAAATGT | GGCATGCCTCTTAGCTAATGTTT |
| ***Meioc*** | TAAGTCGCAGCCATATTTTGCT | GGTTCTGTTAAGAGGTCATGGTG |
| ***Emi2*** | AAGTTTAGGTTCCCCCAATTCTG | CGCCTGCGTAATGTGCATTT |
| ***Ccna2*** | GCCTTCACCATTCATGTGGAT | TTGCTGCGGGTAAAGAGACAG |
| ***Ccnb3*** | AGAGAAACCTGTGATTCAGGAGA | GAAATGCGGCTTTTTGACTGG |
| ***Ccnd3*** | CGAGCCTCCTACTTCCAGTG | GGACAGGTAGCGATCCAGGT |
| ***Ccne2*** | ATGTCAAGACGCAGCCGTTTA | GCTGATTCCTCCAGACAGTACA |
| ***Amhr2*** | GGGGCTTTGGACACTGCTT | GTCTCGGCATCCTTGCATCTC |
| ***Gata4*** | CCCTACCCAGCCTACATGG | ACATATCGAGATTGGGGTGTCT |
| ***Cxcl12*** | TGCATCAGTGACGGTAAACCA | TTCTTCAGCCGTGCAACAATC |
| ***Cxcr4*** | GAAGTGGGGTCTGGAGACTAT | TTGCCGACTATGCCAGTCAAG |
| ***Rara*** | TTCTTTCCCCCTATGCTGGGT | GGGAGGGCTGGGTACTATCTC |
| ***Rhox10*** | GCCCAATGTCGCTTTGGAAG | AGCTGTAGGTTTGCGTGTATTT |
| ***Gapdh*** | AGGTCGGTGTGAACGGATTTG | TGTAGACCATGTAGTTGAGGTCA |
|  |  |  |
