# Supplementary material for: Germline deletion of Cdyl causes teratozoospermia and progressive infertility in male mice
Source: Cell Death Dis. 2019 Mar 8;10(3):229. doi: 10.1038/s41419-019-1455-y (PMC6408431; doi:10.1038/s41419-019-1455-y)
Supplement: Supplementary file 3 — Supplementary Table S3 [file 41419_2019_1455_MOESM3_ESM.doc]

**Supplementary Table 3** Relative expression level from RNA-seq analysis of the sex chromosome-linked genes highly expressed in haploid spermatids ^[28]^.

| Gene symbol | Gene ID |  |  | Sample No. | |  |  |
| --- | --- | --- | --- | --- | --- | --- | --- |
|  |  | Control-1 | Control-2 | Control-3 | cKO-1 | cKO-2 | cKO-3 |
| *Pin4* | 69713 | 14.18 | 17.83 | 13.85 | 15.59 | 16.03 | 21.33 |
| *Ccdc160* | 434778 | 7.02 | 6.54 | 6.67 | 10.94 | 6.87 | 6.74 |
| *Tceal1* | 237052 | 5.14 | 5.3 | 4.09 | 6.38 | 5.06 | 5.72 |
| *Zcchc13* | 75064 | 41.38 | 41.75 | 39.51 | 52.02 | 42.31 | 45.61 |
| *Rnf138rt1* | 74264 | 16.94 | 14.65 | 17.19 | 20.93 | 13.81 | 14.61 |
| *Akap14* | 434756 | 9.12 | 9.73 | 8.99 | 8.91 | 9.5 | 9.17 |
| *Cypt3* | 69361 | 70.92 | 67.87 | 80.98 | 95.23 | 79.26 | 59.33 |
| *Actrt1* | 73360 | 14.03 | 16.05 | 15.58 | 18.05 | 13.07 | 14.01 |
| *Gm732* | 213450 | 9.82 | 10.65 | 11.19 | 12.71 | 12.82 | 11.66 |
| *Dmrtc1a* | 70887 | 23.63 | 24.14 | 19.89 | 31.08 | 21.24 | 25.07 |
| *1700010D01Rik* | 76386 | 156.88 | 171.6 | 131.28 | 173.11 | 175.16 | 155.19 |
| *Cd40lg* | 21947 | 0.05 | 0 | 0.05 | 0 | 0 | 0.1 |
| *Dmrtc1a* | 70887 | 23.63 | 24.14 | 19.89 | 31.08 | 21.24 | 25.07 |
| *Chm* | 12662 | 0.61 | 0.6 | 0.53 | 0.66 | 0.79 | 0.95 |
| *Brwd3* | 382236 | 1.59 | 1.52 | 1.46 | 1.26 | 2.19 | 2.04 |
| *Tex13a* | 67944 | 37.5 | 47.32 | 36.42 | 52.07 | 44.09 | 43.3 |
| *4930519F16Rik* | 75106 | 3.82 | 3.98 | 3.4 | 3.7 | 5.33 | 4.89 |
| *Dusp21* | 73547 | 22.57 | 29.54 | 23.75 | 24 | 21.76 | 15.2 |
| *4933436I01Rik* | 66780 | 20.93 | 27.77 | 21.68 | 15.73 | 30.36 | 25.26 |
| *Zfy2* | 22768 | 3.53 | 3.68 | 3.52 | 2.97 | 4.14 | 3.13 |
