# Supplementary figures and images for: Germline deletion of Cdyl causes teratozoospermia and progressive infertility in male mice
Source: Cell Death Dis. 2019 Mar 8;10(3):229. doi: 10.1038/s41419-019-1455-y (PMC6408431; doi:10.1038/s41419-019-1455-y)

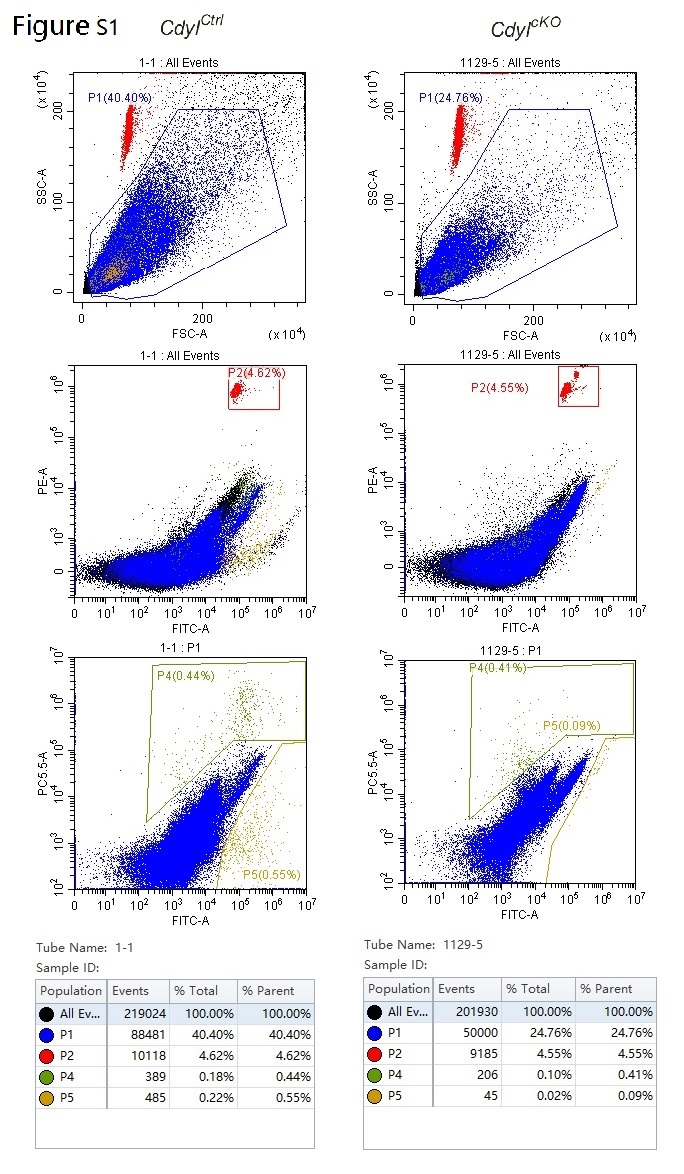

Supplement: Supplementary file 4 — Supplementary Figure S1 [file 41419_2019_1455_MOESM4_ESM.jpg]

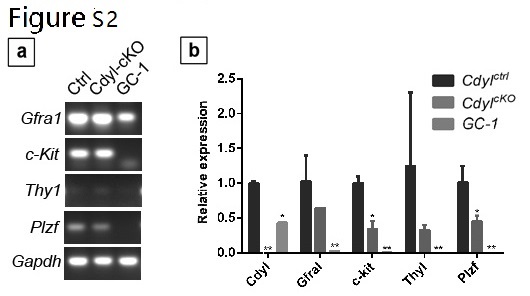

Supplement: Supplementary file 5 — Supplementary Figure S2 [file 41419_2019_1455_MOESM5_ESM.jpg]
