# Supplementary material for: Germline deletion of Cdyl causes teratozoospermia and progressive infertility in male mice
Source: Cell Death Dis. 2019 Mar 8;10(3):229. doi: 10.1038/s41419-019-1455-y (PMC6408431; doi:10.1038/s41419-019-1455-y)
Supplement: Supplementary file 6 — supplemental figure legends [file 41419_2019_1455_MOESM6_ESM.docx]

**CDDIS-18-2970R**

**Germline Deletion of *Cdyl* Causes Teratozoospermia and Progressive Infertility in Male Mice**

**Supplementary Fig. S1 Representative histogram of quantitative flow cytometry for SSCs/ spermatogonia identification.** For bead counting, the beads region was shown as P2, the Thy1+ events were gated as P4, while the c-Kit+ events were gated as P5.

**Supplementary Fig. S2 Relative expression of spermatogenic marker genes in 1-week-old testis and GC-1 cell line.** **a** semi-quantitative RT-PCR; **b** quantitative RT-PCR.
